# Patterns of uptake of HIV testing in sub-Saharan Africa in the pre-treatment era

**DOI:** 10.1111/j.1365-3156.2011.02937.x

**Published:** 2012-07-30

**Authors:** Ide Cremin, Simon Cauchemez, Geoffrey P Garnett, Simon Gregson

**Affiliations:** 1 Department of Infectious Disease Epidemiology, Imperial College LondonLondon, UK; 2 Biomedical Research and Training InstituteHarare, Zimbabwe

**Keywords:** HIV, testing, sub-Saharan Africa

## Abstract

**Objectives:**

To compare nationally representative trends in self-reported uptake of HIV testing and receipt of results in selected countries prior to treatment scale-up.

**Methods:**

Demographic and Health Survey (DHS) data from 13 countries in sub-Saharan Africa were used to describe the pattern of uptake of testing for HIV among sexually active participants. Univariate and multivariate logistic regression were used to analyse the associations between socio-demographic and behavioural characteristics and the uptake of testing.

**Results:**

Knowledge of serostatus ranged from 2.2% among women in Guinea (2005) to 27.4% among women in Rwanda (2005). Despite varied levels of testing, univariate analysis showed the profile of testers to be remarkably similar across countries, with respect to socio-demographic characteristics such as area of residence and socio-economic status. HIV-positive participants were more likely to have tested and received their results than HIV-negative participants, with the exception of women in Senegal and men in Guinea. Adjusted analyses indicate that a secondary or higher level of education was a key determinant of testing, and awareness that treatment exists was independently positively associated with testing, once other characteristics were taken into account.

**Conclusion:**

This work provides a baseline for monitoring trends in testing and exploring changes in the profile of those who get tested after the introduction and scale-up of treatment.

## Introduction

HIV testing and counselling is the entry point for HIV prevention, treatment and care services (UNAIDS 2001; De Cock *et al.* 2003). Provision of and access to testing services has increased rapidly in recent years as provision of anti-retroviral treatment (ART) has expanded dramatically (WHO, UNAIDS & UNICEF 2010). It is important to monitor trends in testing uptake for several reasons. Firstly, it is useful to analyse patterns of uptake of testing with respect to socio-demographic characteristics, as this has implications for equitable access to treatment (Wringe *et al.* 2008; Makwiza *et al.* 2009). Secondly, in terms of prevention, analysing testing patterns with respect to behavioural indicators can help determine whether those individuals most at risk of acquiring or transmitting infection are seeking testing. Finally, understanding the factors affecting the use of testing services is essential for the success of testing programmes in achieving high levels of coverage.

Prior to provider-initiated opt-out testing (UNAIDS and WHO 2007), those going for HIV testing were generally a small self-selecting group of the wider population who chose to access testing services (Obermeyer & Osborn 2007). The profile of those coming forward for testing has not previously been described at a nationally representative level in an African context, except among married men in Uganda (Gage & Ali 2005).

The objectives of this analysis are to: (i) compare nationally representative trends in self-reported uptake of HIV testing and receipt of results in selected countries, and (ii) characterise the profile of participants who report to have tested for HIV.

## Methods

### Survey methods

The Demographic and Health Survey (DHS) programme has been conducting household surveys since 1984 in developing countries (Measure DHS 2009). A two-stage cluster design was used to obtain samples representative at the national level (Demographic and Health Surveys 1996). All women aged 15–49 years and men in the target age range (15–49, 15–54 or 15–59) who were either permanent residents or visitors present on the night before the survey in selected households were eligible to be interviewed. In several surveys, men were selected from a subsample of households.

Since 2001, the DHS and AIDS Indicator Surveys (AIS) have (i) conducted anonymous HIV serotesting, and (ii) included sections on HIV testing and receipt of results in survey questionnaires. All individuals eligible for the individual interview were eligible for anonymous HIV serotesting (Macro International 2007). However, for surveys in which a male subsample was selected, only participants from the households selected for the male subsample were eligible to provide blood for anonymous serotesting. Consenting participants provided blood subsequent to the interview: dried spot samples of capillary blood from a finger prick collected on filter paper. All participants received referrals for free counselling, testing and educational materials, whether or not they consented to anonymous serotesting. In some countries, the DHS programme provided mobile voluntary counselling and testing (VCT) services so that participants could learn their HIV status and receive counselling if they wished.

### Data and analysis

All countries in sub-Saharan Africa which had a DHS or AIS survey; (i) previous to 2006, (ii) which carried out anonymous HIV serotesting, and (iii) which collected self-reported data on HIV testing and results collection, were included in the analysis. Thirteen surveys met these criteria. All of these surveys had taken place before ART roll-out, although the timing of some surveys, such as that in Malawi, coincided with the start of ART programmes. The Zambian and Malian surveys in 2001 were excluded as self-reported data regarding receipt of test results were not available. In the Burkina Faso survey, only male data were analysed as women were not asked about HIV testing.

The outcome variable was defined as having tested and received the result (i.e. knowing one’s HIV status). The self-reported HIV testing data analysed here refer to a wide range of HIV testing services, but not to the anonymous testing carried out through the DHS programme. In the construction of the socio-demographic variables, a wealth index was used to capture poverty level (Rutstein & Johnson 2004).

Univariate logistic regression models were fitted to describe associations between all variables identified *a priori* as characteristics of interest and uptake of testing and collection of results. Following this, a full model was fitted to identify those characteristics which were independently associated with knowing one’s HIV status. Multivariate results are presented and discussed in detail for the following four selected countries: Zimbabwe, Lesotho, Rwanda and Senegal. These four countries were chosen to compare determinants of uptake of testing and receipt of results across different contexts with respect to HIV prevalence and with respect to level of uptake of testing and receipt of results.

All analyses were carried out separately for men and women and restricted to the non-virgin population, as those who have never had sex have had no sexual exposure to HIV and, thus, are likely to have little impetus for seeking HIV testing. Clustering in the survey design was accounted for, and all analyses were carried out using sample weights provided by the DHS (Rutstein & Rojas 2006).

## Results

### Data overview and response rates

Table 1 shows the household and individual response rates for the 13 surveys, and the response rates for HIV testing in the anonymous serosurvey. Overall, household response rates were high and ranged from 95.0% to 99.7%. The individual response rate in each survey was higher among women than among men and ranged from 81.9% among men in Zimbabwe to 98.1% among women in Rwanda. The largest gender differentials in participation were observed in Malawi and Lesotho. Response rates for the anonymous HIV serosurvey were lower than participation rates and ranged from 63.3% among men in Malawi to 97.3% among women in Rwanda. In each survey, the anonymous HIV serosurvey response rate was lower for men than for women.

**Table 1:** Response rates for participation and for the anonymous HIV serosurvey in selected Demographic and Health Surveys (DHS). All data are unweighted, are not restricted to non-virgins, and are compiled from DHS country-specific data and final reports

Country	Year	Survey	Household response rate (%)	Individual response rate (%)	*N* interviewed	*N* eligible for HIV testing	HIV testing response rate (%)
Zimbabwe							
Male	2005–2006	DHS	95.0	81.9	7175	8761	63.4
Female				90.2	8907	9870	75.9
Senegal							
Male	2005	DHS	98.5	86.0	3761	4375	75.5
Female				93.7	14602	5350	84.5
Rwanda							
Male	2005	DHS	99.7	97.2	4820	4959	95.6
Female				98.1	11321	5837	97.3
Guinea							
Male	2005	DHS	99.2	94.5	3174	3360	88.2
Female				97.2	7954	4189	92.5
Ethiopia							
Male	2005	DHS	98.5	89.0	6033	6778	75.4
Female				95.6	14070	7142	83.2
Cote D’Ivoire							
Male	2005	AIS	95.5	87.5	4503	5148	76.3
Female				89.8	5183	5772	79.1
Malawi							
Male	2004	DHS	97.8	85.9	3261	3797	63.3
Female				95.7	11698	4071	70.4
Lesotho							
Male	2004	DHS	95.2	84.6	2797	3305	68.0
Female				94.3	7095	3758	80.7
Cameroon							
Male	2004	DHS	97.6	93.0	5280	5676	89.8
Female				94.3	10656	5703	92.1
Tanzania							
Male	2003–04	AIS	98.5	91.3	5659	6196	77.0
Female				95.9	6863	7154	83.5
Burkina Faso							
Male	2003	DHS	99.4	90.5	3605	3984	85.8
Kenya							
Male	2003	DHS	96.3	85.5	3578	4183	70.3
Female				94.0	8195	4303	76.3
Ghana							
Male	2003	DHS	98.7	93.8	5015	5345	80.0
Female				95.7	5691	5949	89.3

AIS, AIDS Indicator Surveys.

The overall household response rate is given for surveys in which a male subsample was used. In Rwanda, Guinea, Ethiopia, Lesotho, Cameroon and Kenya, all men in 50% of households were sampled. In Senegal, Malawi and Burkina Faso, all men in 33% of households were sampled. Thus, for these countries, the number of women eligible for the anonymous HIV serosurvey is lower than the number interviewed, as only women in the households selected for the male subsample were eligible for the anonymous HIV serosurvey. In Ethiopia, only women in households selected for the male subsample were administered questions on prior HIV testing.

### Uptake overview

**Figure 1:**
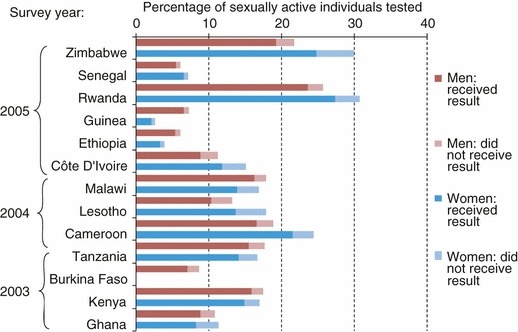
Reported uptake of testing and results collection among men and women in selected countries.

Levels of reported uptake of HIV testing and collection of results varied widely between countries (Figure 1). Uptake was highest among women in Rwanda, at 27.4% in 2005. The lowest levels of testing and collection of results were observed in Ethiopia and in West African countries, Guinea and Senegal in particular. However, not all West African countries analysed had low uptake; for example, Cameroon had the third highest overall percentage of persons tested. Generally, reported receipt of results among those tested was high and ranged from 75.2% to 98.3%. Gender differentials in uptake were observed in many countries; for example, in Zimbabwe, Rwanda, Côte D’Ivoire, Lesotho and Cameroon, women were more likely to have tested and received their results; whereas, in Guinea and Ethiopia, uptake of testing and results collection was higher among men.

When asked about the last time they were tested, a considerable proportion of those who had tested reported to have done so within the last 12 months (mean 46%), possibly reflecting the scale-up of testing services in these countries. Furthermore, several surveys asked respondents whether they would like to be tested (i.e. go for VCT). In these surveys, the majority of participants who reported not to have tested responded that they would like to be tested (mean 69%), indicating a considerable unmet demand for HIV testing services.

### Characteristics associated with knowing one’s HIV status

#### HIV infection

**Figure 2:**
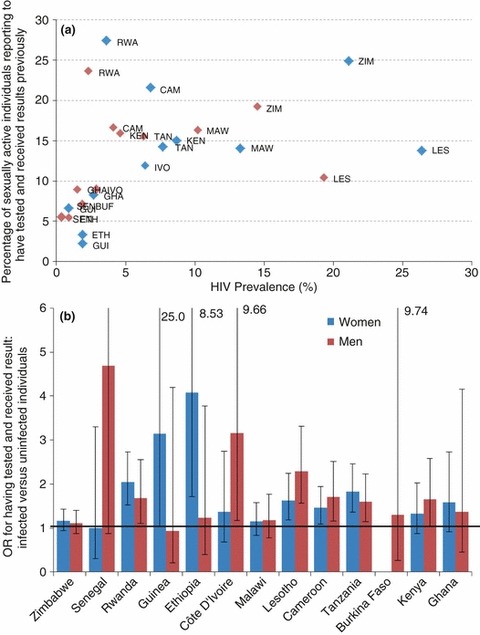
The relationship between HIV infection and knowledge of HIV status. (a) Uptake of HIV testing and receipt of results with respect to HIV prevalence among women and men in selected countries. Each point on the scatter plot represents a national level estimate based on data from the same Demographic and Health Survey/AIDS Indicator Survey. Blue, women; red, men. (b) Odds ratios and corresponding 95% confidence intervals for knowing one’s HIV status with respect to HIV status (positive *vs.* negative), for men and women. BUF, Burkina Faso; CAM, Cameroon; ETH, Ethiopia; GHA, Ghana; GUI, Guinea; IVO, Côte D’Ivoire; KEN, Kenya; LES, Lesotho; MAW, Malawi; RWA, Rwanda; SEN, Senegal; TAN, Tanzania; ZIM, Zimbabwe.

At a country level, no clear relationship between HIV prevalence and knowledge of HIV status was observed (Figure 2a). For example, in 2005, HIV prevalence among women in Guinea was 1.9% and knowledge of HIV status was very low at 2.2%, whereas in Rwanda, although HIV prevalence among women was relatively low at 3.6%, knowledge of HIV status was high at 27.4%. The correlation coefficients for women and men were 0.42 (*P* < 0.0001) and 0.35 (*P* < 0.0001), respectively, indicating a weak but significant positive correlation. However, although no clear linear relationship was observed, there is a logarithmic relationship, with Rwanda and Lesotho as outliers.

At an individual level, HIV-positive participants were more likely to have tested and received their results than uninfected participants, with the exception of women in Senegal and men in Guinea (Figure 2b). This association was statistically significant among men and women in Rwanda, Lesotho, Cameroon and Tanzania, men in Côte D’Ivoire and Kenya, and women in Guinea and Ethiopia. In countries with relatively low levels of HIV prevalence and low levels of testing and receipt of results, such as Ethiopia and those in West Africa (i.e. Senegal, Guinea, Côte D’Ivoire, Burkina Faso and Ghana), there is very large uncertainty around the odds ratios (ORs).

### Socio-demographic characteristics

**Figure 3:**
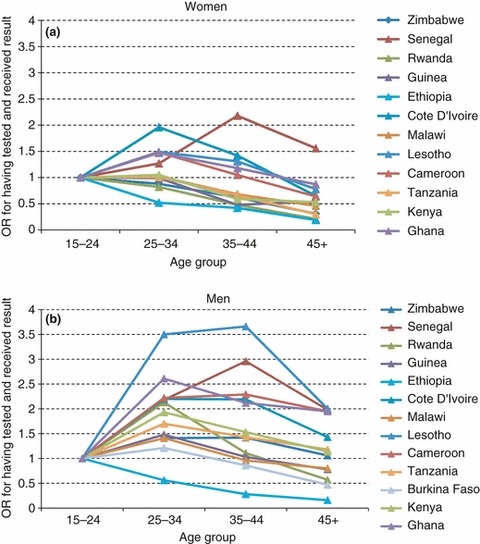
Odds ratios for knowing one’s HIV status by 10-year age group, among women (a) and men (b).

Among women, older age group was significantly negatively associated with knowing one’s HIV status in all surveys, except in Senegal, where uptake was lowest amongst 15–25 year olds (Figure 3a). In Cote d’Ivoire, Ghana, Lesotho and Cameroon, uptake was higher among women aged 25–34 years than among those aged 15–24 years, but subsequently declined in older age groups. Among men, age was also significantly associated with knowing one’s HIV status, except in Guinea. The general trend was an increase in uptake from 15 to 24 years up to 25–34 years, followed by a plateau or slight decrease and then a decline in the oldest age group, with the exception of Ethiopia (Figure 3b). This trend was most apparent in Lesotho. In general, men seeking testing were older than women seeking testing, across these thirteen countries.

**Figure 4:**
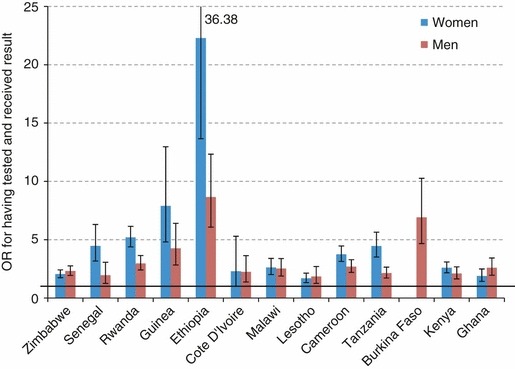
Odds ratios and corresponding 95% confidence intervals for knowing one’s HIV status by place of residence (urban *vs.* rural), for women and men.

The majority of participants lived in rural areas, where uptake was significantly lower relative to those living in urban areas, in all surveys. Among women, the ORs for uptake by residence (urban *vs.* rural) ranged from 1.7 in Lesotho to 22.3 in Ethiopia (Figure 4). Similar trends were observed among men, where the odds of uptake in urban areas were between 1.9 and 8.7 times greater than in rural areas. Uptake was greatest in urban Rwanda at 58.6% and 41.9% among women and men, respectively. In addition to large differentials with regard to place of residence, strong regional differences within countries were also observed (i.e. much heterogeneity within rural areas, data not shown).

**Figure 5:**
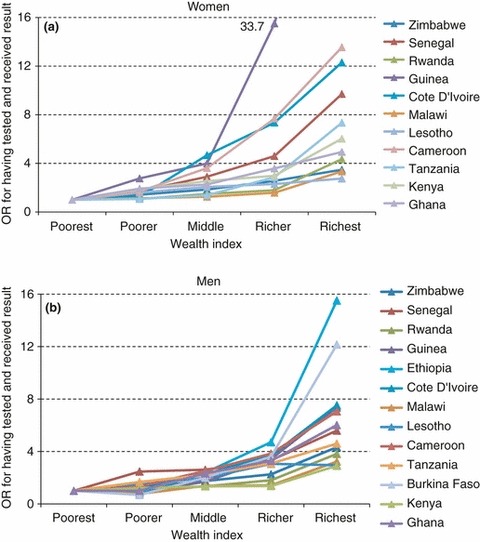
Odds ratios for knowing one’s HIV status by wealth quintile, for women (a) and men (b).

Among women, a strong significant positive association between wealth quintile and knowing one’s HIV status was observed (Figure 5a). Those women in the richest quintile were more likely to know their HIV status than those in the poorest quintile (OR range, 2.8–33.7). Odds ratios were not calculated for Ethiopia as 81.1% of women who tested and received their result were in the richest wealth quintile. A similar trend was observed for men with respect to wealth index. Although statistically significant, the differentials in uptake were generally not as great as those for women. The odds of testing and receiving results were between 2.9 and 15.5 times greater among those in the richest quintile as compared to the poorest (Figure 5b).

Across all countries, large differentials in uptake with respect to level of education were observed among both men and women, with uptake being greatest among those with higher level education. Overall, with respect to socio-demographic characteristics, the trends were remarkably similar across all countries analysed, for both men and women.

### Sexual behaviour, experience of AIDS and stigma

Reported uptake of testing and results collection varied with respect to various indicators of sexual behaviour, including lifetime partners, ever paying for sex (asked to men only), extra-marital partners in the last year and condom use at last intercourse. Those who reported that they did not know someone who has AIDS or died of AIDS were much less likely to report having tested and received their results. This trend was consistent across all countries. Uptake of testing and results collection was significantly lower among those expressing stigmatising attitudes towards HIV. This was true across all countries for both men and women.

### Multivariate analysis of characteristics associated with knowing one’s HIV status

Table 2 shows the adjusted ORs for the hypothesised determinants of testing and results collection, for women and men in four selected countries. Although univariate analyses found a positive HIV status to be significantly associated with knowing one’s HIV status among men and women in Lesotho and Rwanda (Figure 2b), adjusted analyses showed that being infected was only independently associated with knowing one’s HIV status among men and women in Lesotho.

**Table 2:** Adjusted odds ratios for hypothesised determinants of HIV testing and results collection for men and women in selected countries

		Zimbabwe		Lesotho	
			
		Women	Men	Women	Men
					
Determinants		AOR (95% CI)	AOR (95% CI)	AOR (95% CI)	AOR (95% CI)
HIV status: positive *vs.* negative		1.10 (0.86–1.40)	1.10 (0.83–1.47)	**1.50 (1.06–2.12)**	**1.85 (1.19–2.87)**
Demographic characteristics					
Age group					
15–24		1	1	1	1
25–34		**0.72 (0.59–0.87)**	1.52 (1.00–2.30)‡	1.11 (0.71–1.71)	**4.27 (1.94–9.39)**
35–44		**0.52 (0.39–0.69)**	1.37 (0.83–2.26)	0.95 (0.60–1.49)	**4.27 (1.72–10.60)**
45+		**0.30 (0.18–0.50)**	1.42 (0.81–2.49)	0.82 (0.43–1.57)	**3.52 (1.33–9.30)**
Education					
None		0.88 (0.45–1.70)	1.63 (0.37–7.07)	0.47 (0.14–1.57)	0.66 (0.36–1.21)
Primary		1	1	1	1
Secondary		**1.61 (1.28–2.04)**	**1.76 (1.24–2.50)**	0.99 (0.70–1.41)	**1.80 (1.10–2.95)**
Higher		**4.13 (2.37–7.21)**	**3.16 (1.82–5.50)**	1.49 (0.50–4.38)	2.50 (0.62–10.12)
Marital status					
Married		1	1	1	1
Never married		0.70 (0.36–1.36)	0.73 (0.38–1.41)	0.84 (0.44–1.61)	0.91 (0.42–2.01)
Previously married		0.96 (0.51–1.80)	**0.22 (0.07–0.65)**	1.20 (0.64–2.27)	1.58 (0.42–5.91)
Residence					
Urban *vs*. rural		1.19 (0.75–1.87)	1.14 (0.75–1.73)	1.48 (0.99–2.20)‡	1.22 (0.70–2.14)
Poverty					
Richest quintile *vs*. poorest		**2.24 (1.29–3.90)**	**2.63 (1.35–5.13)**	1.41 (0.75–2.65)	1.11 (0.47–2.63)
Sexual health & behaviour					
Condom use at last intercourse^a^: yes *vs*. no		0.86 (0.59–1.26)	**1.60 (1.14–2.25)**	1.37 (0.90–2.08)	**1.81 (1.18–2.78)**
Extra-marital partners in last year: yes *vs*. no		1.00 (0.60–1.66)	1.16 (0.70–1.92)	0.80 (0.48–1.33)	**0.54 (0.32–0.90)**
Lifetime partners					
Women	Men				
1	1	1	1	–	1
2	2	0.94 (0.73–1.22)	0.71 (0.47–1.05)	–	1.23 (0.51–2.99)
>2	3	0.96 (0.71–1.30)	**0.64 (0.41–0.99)**	–	1.51 (0.64–3.54)
	4		**0.48 (0.28–0.81)**		0.78 (0.30–2.05)
	>4		0.81 (0.55–1.17)		1.28 (0.61–2.70)
Ever paid for sex (men only): yes *vs*. no		–	1.02 (0.48–2.14)	–	
Had an STD in last year: yes *vs*. no		1.20 (0.77–1.87)	1.33 (0.64–2.77)	0.97 (0.44–2.16)	1.85 (0.65–5.28)
Treatment awareness: yes *vs*. no/unsure		**1.84 (1.48–2.29)**	1.22 (0.92–1.62)	**1.64 (1.15–2.34)**	**1.64 (1.07–2.50)**
Expression of stigma & experience of AIDS					
People with AIDS should be ashamed: agree *vs*. disagree/don’t know		1.13 (0.91–1.41)	0.96 (0.72–1.29)	–	–
AIDS bereavement: yes *vs*. no		1.08 (0.87–1.34)	**1.32 (1.05–1.65)**	**2.15 (1.59–2.91)**	0.95 (0.57–1.58)
HIV status: positive *vs*. negative		1.18 (0.71–1.94)	0.99 (0.54–1.82)	0.46 (0.03–8.26)	1.62 (0.19–13.64)
Demographic characteristics					
Age group					
15–24		1	1	1	1
25–34		**0.61 (0.47–0.80)**	0.83 (0.56–1.23)	0.74 ( 0.28–1.95)	**17.69 (1.91–164.20)**
35–44		**0.33 (0.24–0.44)**	**0.35 (0.23–0.54)**	2.33 (0.95–5.69)	**22.19 (1.80–273.00)**
45+		**0.13 (0.07–0.23)**	**0.20 (0.13–0.32)**	1.15 (0.41–3.25)	**19.01 (1.69–213.63)**
Education					
None		0.81 (0.62–1.05)	0.77 (0.56–1.06)	0.91 (0.37–2.22)	0.75 (0.18–3.17)
Primary		1	1	1	1
Secondary		**1.65 (1.15–2.36)**	**1.45 (1.03–2.04)**	**2.21 (1.12–4.36)**	**4.04 (1.06–15.46)**
Higher		7.73 ( 0.85–70.45)	**4.47 (1.75–11.43)**	**50.62 (14.35–178.60)**	**8.72 (2.08–36.53)**
Marital status					
Married		1	1	1	1
Never married		0.66 (0.31–1.40)	**0.43 (0.20–0.95)**	0.56 (0.05–5.78)	0.96 (0.10–9.42)
Previously married		0.78 ( 0.30–2.05)	2.35 (0.72–7.71)	**4.70 (1.95–11.34)**	
Residence					
Urban *vs*. rural		**3.68 (2.68–5.06)**	**1.77 (1.28–2.45)**	1.73 (0.71–4.20)	1.09 (0.37–3.26)
Poverty					
Richest quintile *vs*. poorest		**1.61 (1.09–2.37)**	**1.93 (1.29–2.88)**	3.24 (0.90–11.64)	0.52 (0.08–3.39)
Sexual health & behaviour					
Condom use at last intercourse: yes *vs*. no		0.92 (0.51–1.66)	**2.00 (1.05–3.78)**	2.04 (0.85–4.92)	0.59 (0.07–4.96)
Extra-marital partners in last year: yes *vs*. no		0.79 (0.42–1.49)	0.69 (0.40–1.19)	0.93 (0.50–1.72)	1.07 (0.32–3.54)
Lifetime partners					
Women	Men				
1	1	1	1	–	–
2	2	1.06 (0.81–1.39)	**1.46 (1.08–1.98)**	–	–
>2	3	0.84 (0.56–1.26)	1.40 (0.98–2.00)	–	–
	4		**1.99 (1.32–3.00)**		–
	>4		**1.74 (1.16–2.60)**		–
Ever paid for sex (men only): yes *vs*. no		–	1.02 (0.65–1.61)	–	4.27 (0.34–53.17)
Had an STD in last year: yes *vs*. no		1.30 (0.54–3.14)	1.86 (0.74–4.69)	0.54 (0.12–2.40)	b
Treatment awareness: yes *vs*. no/unsure		**2.99 (2.06–4.35)**	**1.99 (1.23–3.21)**	1.73 (0.98–3.06)*	1.02 (0.31–3.32)
Expression of stigma & experience of AIDS					
People with AIDS should be ashamed: agree *vs*. disagree/don’t know		**0.75 (0.60–0.93)**	0.90 (0.70–1.16)	0.60 (0.34–1.06)	0.55 (0.21–1.41)
AIDS bereavement: yes *vs*. no		1.26 (0.93–1.71)	**1.98 (1.25–3.13)**	**4.01 (1.84–8.72)**	1.52 (0.56–4.10)

AOR, adjusted odds ratio.

In Lesotho, data on ‘People with AIDS should be ashamed’ were not available for neither men nor women, and lifetime partners data were not available for women, and ever paid for sex was not available for men. In Senegal, data on lifetime partners were not available for neither men nor women. Bold type indicates differences significant at the 95% level. For men in Senegal, the two poorest wealth quintiles were combined into a single category for the reference group, as there were too few respondents who had tested in the poorest quintile.

* Indicates significant in univariate analysis (*P* < 0.05).

‡ borderline significance.

a Condom use refers to last partner, rather than specifically last sex, for men in Lesotho.

b Variable dropped owing to small sample size in comparison group (those reporting an STD in last year).

Univariate analyses also indicated age group to be a significant determinant of knowing one’s HIV status among men and women in each of these four countries. However, this association was no longer significant in multivariate analyses among women in Lesotho and Senegal, and men in Zimbabwe. In general, trends in uptake with respect to age differed for men and women, with uptake being greatest among younger women, but lowest among younger men, except for men in Rwanda. However, divergent trends were observed among men, as the ORs for uptake among the oldest age group (45 years and older) as compared to the youngest (15–24 years) ranged from 0.2 for men in Rwanda to 19.5 for men in Senegal.

Increased odds of knowing one’s HIV status were consistently observed with increasing level of education for both men and women, in all four countries. Although reporting a tertiary level of education was infrequent, it was strongly associated with increased HIV testing and results collection (OR range: 1.5–50.6). Living in an urban area was also associated with increased uptake in all four populations, but only remained significant in adjusted analyses for women and men in Rwanda. Similarly, an increasing level of wealth was strongly associated with increased uptake in the univariate analysis, but this association only remained a significant determinant in adjusted analyses among men and women in Zimbabwe and in Rwanda.

Generally, there was mixed evidence for associations between marital status, behavioural indicators and knowledge of one’s HIV status. There was no evidence to suggest that infrequently reported, but high-risk behaviours, such as paying for sex among men, were independently associated with knowing one’s HIV status. Reporting having had an STD in the last year generally showed non-significant associations with increased uptake of testing and receipt of results.

An awareness of the existence of treatment was independently associated with increased uptake among men and women in all four countries, with the exception of men in Senegal. Those who agreed with the stigmatising attitude ‘people with AIDS should be ashamed’ were less likely to know their HIV status, with the exception of women in Zimbabwe. However, this association only remained significant among women in Rwanda. Knowing someone who has, or has died from, AIDS was associated with increased uptake among men and women in all four countries, with the exception of men in Lesotho.

Overall, univariate analyses showed remarkably similar trends across countries and many of these associations persisted at the multivariate level. Level of education and awareness that treatment for HIV exists were consistently independently associated with knowing one’s HIV status across countries, for both men and women.

## Discussion

These analyses provide a cross-sectional illustration of the varied levels of uptake of HIV testing, and results collection in thirteen African countries, each of which represents a very different epidemiological context in terms of HIV prevalence as well as varied provision of and access to testing services. Overall, knowledge of serostatus was limited during this period. Despite the considerable variations in the percentage of participants who knew their HIV status, from 2.2% to 27.4%, univariate analyses indicated that the socio-demographic profiles of testers (well-educated, wealthier participants living in urban areas) were remarkably similar across countries. However, in detailed multivariate analyses for a smaller sub-set of countries, many of these associations did not persist as significant determinants of testing. Despite large differentials in uptake with respect to level of wealth and living in an urban area *vs.* a rural area, the results from adjusted models indicate that a secondary or higher level of education is a key determinant of testing and awareness that treatment exists is associated with testing, once other possible factors have been taken into account. However, the association between awareness that treatment exists and uptake of testing may reflect the fact that individuals receive information regarding treatment during pre- and post-test counselling.

The socio-demographic and behavioural-risk profile of individuals who test for HIV has implications for HIV prevention and equitable access to treatment. In terms of prevention, these analyses showed the behavioural profile of those coming forward for testing to be mixed, although this may reflect differences in reporting bias across countries. It is encouraging that testing is not preferentially attracting a ‘worried-well’ low-risk group, but, on the other hand, evidence that some high-risk behaviours, such as transactional sex, are not prompting testing may be of concern. In terms of testing as a means of identifying those in need of treatment, an over-representation of infected participants is encouraging. However, in terms of equitable access to treatment, the emergence of those with little or no education, who live in rural areas in the poorest households and who are unaware of treatment, as an under-represented group amongst those accessing testing, may also be of concern.

The strongest independent predictor of testing among both sexes is level of education. For example, when compared to those with primary education, women in Senegal who had a tertiary education had 50 times the adjusted odds of having tested. Education has been found to be a key determinant of uptake of testing in many other settings (Gage & Ali 2005; Matovu *et al.* 2005; Hutchinson & Mahlalela 2006; Weiser *et al.* 2006; Sherr *et al.* 2007; Wringe *et al.* 2008). This indicates that there may be a need to increase the awareness of the benefits of counselling and testing in a way that is accessible to those with little or no formal education.

Increased uptake of testing among those living in urban areas, as compared to rural areas, may be due to greater availability of services in the former (i.e. initially setting up sites in urban areas and then gradually extending to rural areas). The extent to which uptake increases with rising level of wealth is striking, given that HIV counselling and testing services are generally either inexpensive or provided for free. However, in rural areas, transport costs may be a barrier to accessing testing services for some individuals.

Owing to the cross-sectional nature of the data, a caveat regarding uptake with respect to HIV status is that some participants may have seroconverted between the time they tested and when the DHS survey, and anonymous testing, was carried out. However, the majority of participants reported to have tested quite recently (within the last year or 2 years), which would reduce this misclassification. Moreover, non-response for participation in the DHS HIV testing, among men in particular, may bias these results with respect to HIV status (Reniers & Eaton 2009). Given the cross-sectional nature of the data, it was not possible to establish the temporal sequence between reported behaviour, experience of AIDS or stigma, and knowing one’s HIV status.

In this analysis, it was not possible to test the hypothesis that individuals, if infected, may only seek testing once they are showing symptoms of advanced HIV infection and suspect that they may be infected. However, a population-based study in Rakai, Uganda, has found evidence that counselling and testing is associated with reported illness and potential AIDS-related symptoms among women, but not among men (Nyblade *et al.* 2001). It was also not possible to test the hypothesis that death of one’s spouse may prompt an individual to seek testing. Although knowing someone who has, or has died from, AIDS was associated with increased uptake, data on the relationship to that person were not available. However, in some settings, this indicator may be capturing denial of AIDS (whereby individuals deny that they know anyone who has, or has died from, AIDS), rather than experiencing bereavement. For example, in Lesotho, only 28% of women and 29% of men reported knowing someone with, or who had died from, AIDS, despite Lesotho having a high level of HIV prevalence.

A clear linear relationship between the uptake of testing and HIV prevalence at a national level was not apparent. Rwanda with an HIV prevalence of <5% had the highest level of uptake overall, whereas Lesotho, with the highest HIV prevalence had a much lower uptake.

The provision of testing services (i.e. supply) and the demand for testing both play an important role in determining the level of uptake of testing. In terms of supply, a key determinant of testing, not included in these analyses, is availability of and access to testing services. Ideally, the denominator for these analyses would be those who have access to testing services. Recent data indicate much heterogeneity in the level of service provision across countries, even when using the comparative measure of number of adults per testing and counselling facility (WHO, UNAIDS *et al.* 2010). In terms of demand, the majority of participants reported that they would like to be tested. However, even when services are available, reported willingness to test does not necessarily translate into uptake of testing (Wringe *et al.* 2008). Availability of treatment increases demand for testing, as in the absence of treatment, knowing that one is infected may be viewed as futile (Day *et al.* 2003). Future analyses of testing uptake should focus on analysing the extent to which increasing availability of (i) testing services and (ii) ART, influences the profile of those who test.

Current levels of uptake in these countries are considerably higher, as testing services continue to be scaled up in the context of the shift towards provider-initiated opt-out testing and increased access to treatment (WHO, UNAIDS *et al.* 2010). Monitoring the socio-demographic and behavioural characteristics of testers will provide useful information for the evaluation of these programmes. This work provides a baseline for monitoring trends in testing and exploring changes in the profile of those who get tested as provision of services increase.
